# High-Cycle, Push–Pull Fatigue Fracture Behavior of High-C, Si–Al-Rich Nanostructured Bainite Steel

**DOI:** 10.3390/ma11010054

**Published:** 2017-12-29

**Authors:** Jing Zhao, Honghong Ji, Tiansheng Wang

**Affiliations:** 1 State Key Laboratory of Metastable Materials Science and Technology, Yanshan University, Qinhuangdao 066004, China; zjjysu@163.com (J.Z.); jhh123ykl@163.com (H.J.); 2 National Engineering Research Center for Equipment and Technology of Cold Strip Rolling, Yanshan University, Qinhuangdao 066004, China

**Keywords:** steel, nanostructured bainite, fatigue, crack propagation

## Abstract

The high-cycle, push–pull fatigue fracture behavior of high-C, Si–Al-rich nanostructured bainitic steel was studied through the measurement of fatigue limits, a morphology examination and phase composition analysis of the fatigue fracture surface, as well as fractography of the fatigue crack propagation. The results demonstrated that the push–pull fatigue limits at 10^7^ cycles were estimated as 710–889 MPa, for the samples isothermally transformed at the temperature range of 220–260 °C through data extrapolation, measured under the maximum cycle number of 10^5^. Both the interior inclusion and the sample surface constituted the fatigue crack origins. During the fatigue crack propagation, a high amount of secondary cracks were formed in almost parallel arrangements. The apparent plastic deformation occurred in the fracture surface layer, which induced approximately all retained austenite to transform into martensite.

## 1. Introduction

Most applications of structural materials involve cyclic loading. Consequently, the fatigue behavior should be studied. Many studies have been carried out on the fatigue behavior of bainite/martensite (B/M) dual steels [1,2,3,4,5,6,7,8,9]. Zhao et al. [1,2] reported that the microstructure had a significant effect on the very-high-cycle fatigue (VHCF) behavior of B/M dual-phase steels [1], and that microalloying with Nb was a potential approach to enhance the VHCF properties [2]. Yu et al. [3] reported that the VHCF properties of B/M steels were less sensitive to inclusions compared to the tempered martensite steels. Also, the amount of retained austenite had a low effect on the VHCF properties. In contrast, in highly clean steel, soft-structure-induced fatigue crack was a type of primary VHCF failure mechanism of Nb-microalloyed, carbide-free, bainite/martensite, dual-phase steel [4]. Recently, the high-cycle fatigue behavior of nanostructured bainitic steel with an excellent strength–toughness combination has been paid significant attention [10,11,12]. Peet et al. [10] studied the axial fatigue behavior of nanostructured bainitic steel and indicated that the fatigue limit for no failure in 10^7^ cycles was 855 MPa, which was estimated through the data extrapolation of the 10^5^ maximum cycle number. Also, it was considered that the samples tested at lower loads survived 10^5^ cycles, which possibly led the extrapolation to be a conservative estimation of the fatigue limit. The authors’ group reported that the bending fatigue limit for no failure at 10^7^ cycles of a high-C, Si–Al-rich, nanostructured bainitic steel was determined not only by the hardness or strength, but also by the microstructure [11]. Solano-Alvarez et al. [12] studied the degradation of nanostructured bainitic steel under rolling contact fatigue. It was demonstrated that the damage mechanism was ductile void formation at the interfaces, followed by the growth and coalescence into high-sized voids that lead to fractures along the softer phase direction. In the aforementioned study, the three-point bending was utilized to measure the fatigue limit, which was sensitive to the sample surface and of lower relevance to the fatigue behavior evaluation of the samples. Therefore, in this work, the push–pull, high-cycle fatigue behavior of the high-C, Si–Al-rich nanostructured bainitic steel was studied, which involved the measurement of fatigue limits, a morphology examination and phase composition analysis of the fatigue fracture surface, as well as the fractography of the fatigue crack propagation. In addition, in view of the fact that the microstructure in the steel with metastable austenite did affect the low-cycle fatigue failure feature [13], the retained austenite behavior in the nanostructured bainitic steel during the push–pull, high-cycle fatigue was also addressed.

## 2. Materials and Methods

**Figure 1:**
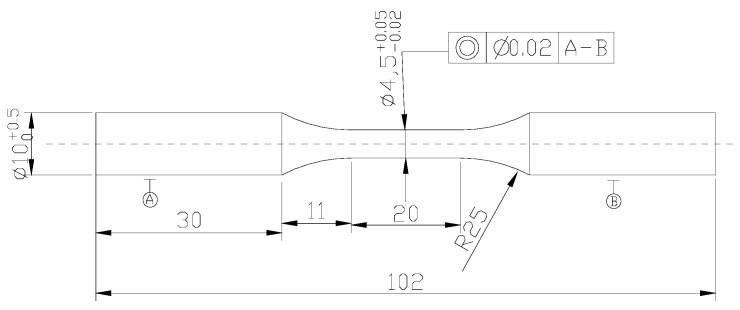
Detailed drawing of push–pull fatigue sample.

The high-C, Si–Al-rich steel used was smelted with a vacuum induction furnace (Shanghai Institute of Optics and Fine Mechanics, Shanghai, China) and cast into a 170 mm in diameter ingot, followed by hot rolling into a ~20 mm in thickness plate. The steel chemical composition was 0.80C–1.59Si–0.81Cr–1.35Mn–0.89W–1.47Al–0.01P–0.0055S (wt %). The plate was spheroidized through heating at 810 °C for 100 min and furnace cooled down to 720 °C, where it was retained for 5 h. Following this, it was air cooled down to ambient temperature. Subsequent to spheroidization, the plate was machined into rectangular blocks of 17 mm × 20 mm × 110 mm in dimensions, leading the length direction to be parallel to the rolling direction. A group of blocks was austenitized at 1000 °C for 30 min and rapidly immerged into a salt bath mixture of NaNO_2_ and KNO_3_ of 1:1 in the weight ratio to isothermally transform at 220 °C for 24 h, 240 °C for 12 h and 260 °C for 4 h (designated as A1, A2 and A3, respectively). The tensile properties of the isothermally-transformed samples were measured on an MTS Landmark Servohydraulic Test System (MTS, Washington, DC, USA), where the plate tensile samples were of 30 mm × 10 mm × 2.8 mm in gauge dimensions. The hardness was measured on an FM-ARS 9000 microhardness tester (TOSHIBA TELI CORPORATION, Tokyo, Japan) at a load of 0.5 kg. Push–pull, high-cycle fatigue tests of the isothermally transformed samples were conducted on an MTS Landmark Servohydraulic Test System (MTS, Washington, DC, USA) according to Chinese National Standard GB/T 3075–2008, with the loading frequency of 20 Hz and the stress ratio of 0.1. Each stress cycle consisted of two steps: the minimum tensile stress followed by a linear ramp to the maximum stress and a ramp down to the minimum stress, namely the tapered ramp profile. The maximum stresses of 1.2, 1.3, 1.4, 1.5 and 1.6 GPa were selected. A detailed drawing of the fatigue sample shape is presented in Figure 1. The surface roughness was 0.4 μm.

The microstructures of the isothermally transformed samples were examined through optical microscopy (OM, Axiover 200MAT, Zeiss, Oberkochen, Germany), transmission electron microscopy (TEM, JEM-2010, JEOL, Musashino, Japan) and X-ray diffraction (XRD, D/max–2500/PC, Rigaku, Tokyo, Japan). The samples utilized for OM and XRD were mechanically ground with waterproof abrasive papers (Blue Dragon, Shanghai, China), polished with diamond paste (Flashing, Shanghai, China) and etched with a 3% Nital solution (ZIBO AO CHEMICAL Co., Ltd., Zibo, China). The samples for TEM examination were sliced into pieces of ~0.6 mm in thickness through wire electrode discharging and mechanically ground down to 30 μm in thickness with waterproof abrasive papers. Also, the samples were thinned to perforation on a TenuPol-5 twin-jet electropolishing device (Struers, Copenhagen, Denmark), with an electrolyte composed of 7% perchloric acid and 93% of glacial acetic acid at ambient temperature and at 40 V. The volume fraction of the retained austenite (VRA) was measured through XRD *θ*–2*θ* step scanning of 0.02° in step width and 2 s of counting time. The fatigue fracture surfaces were examined with an scanning electron microscope (SEM, Hitachi S–3400 and Hitachi S–4800, Hitachi, Tokyo, Japan). The inclusions on the fracture were identified with an X-ray energy dispersive spectrometer (EDS), equipped to SEM.

## 3. Results and Discussion

### 3.1. Microstructure and Mechanical Properties

**Figure 2:**
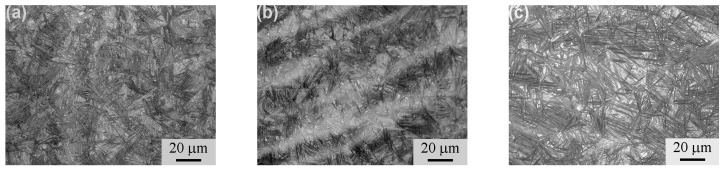
OM micrographs of isothermally-transformed samples. (**a**) A1; (**b**) A2; (**c**) A3.

**Figure 3:**
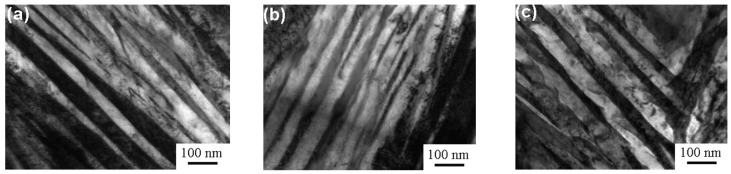
TEM micrographs of isothermally-transformed samples. (**a**) A1; (**b**) A2; (**c**) A3.

Figure 2 presents the OM micrographs of the isothermally-transformed samples. Acicular bainite and bright blocky retained austenite were formed in the samples. The length and thickness of the acicular bainite, as well as the size and the fraction of the retained austenite increased as the isothermal temperature increased. Figure 3 displays the TEM micrographs of the isothermally-transformed samples, presenting the bainitic ferrite laths and retained austenite films. The bright laths are bainitic ferrite and the dark films between them are retained austenite. Cementite precipitates were not observed through the extensive TEM examinations. The mean bainitic ferrite lath thickness (*t*) was determined through the mean lineal intercept *L_T_* measurement in a direction normal to the lath length and stereologically corrected in terms of *L_T_* = *πt*/2 [14]. The *t* values were determined as 27 ± 7, 33 ± 4 and 49 ± 8 nm for the A1, A2 and A3 samples, respectively. Therefore, it could be considered that the carbide-free, nanostructured bainite was prepared by isothermal transformation. The mechanical properties of all the samples were measured and are summarized in Table 1. As the isothermal transformation temperature increased, the impact toughness (*a_KU_*) and elongation (El) increased, whereas the hardness, the ultimate tensile strength (UTS) and the yield strength (YS) presented the opposite trend.

### 3.2. Fatigue Limits

**Figure 4:**
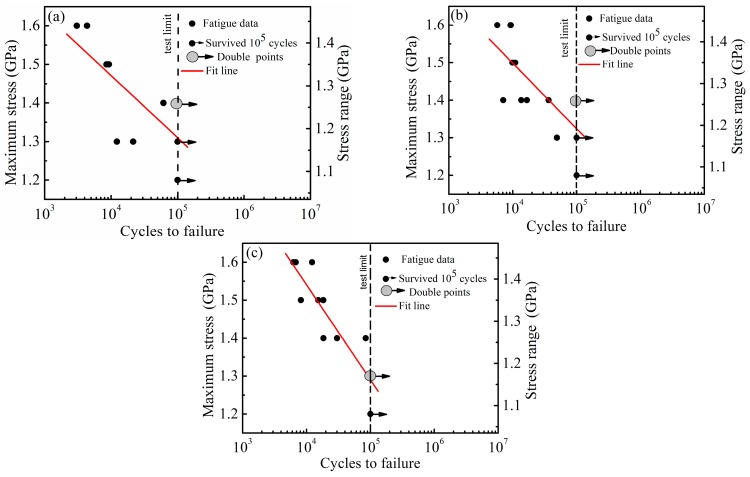
*S*–*N* curves obtained by high-cycle, push–pull fatigue tests of isothermally transformed samples. (**a**) A1; (**b**) A2; (**c**) A3.

Figure 4 presents the *S*–*N* curves obtained by the high-cycle, push–pull fatigue tests of the A1, A2 and A3 samples. The circles represented the sample break data prior to the cycle number of 10^5^, where the gray circles represent the two overlapping data, whereas the circles with arrows represented the unbroken sample data up to 10^5^ cycles. Through regression analysis and extrapolation, the fatigue limits at 10^7^ cycles were estimated as 889, 877 and 710 MPa for the A1, A2 and A3 samples, respectively. The fatigue limits are significantly lower compared to the high-cycle bending fatigue case [11], due to various loading forms. Furthermore, the push–pull fatigue limit increased as the UTS or hardness increased. Compared to the results of Peet et al. [10], the A1 and A2 samples had slightly higher fatigue limits, despite having lower hardness and UTS. This might be attributed to the fact that the steel in the present work was vacuum melted and the steel in the work of Peet et al. [10] was air melted. The cleanliness of the steel melted in vacuum was higher compared to the steel melted in the air.

### 3.3. Fatigue Fracture Analysis

**Figure 5:**
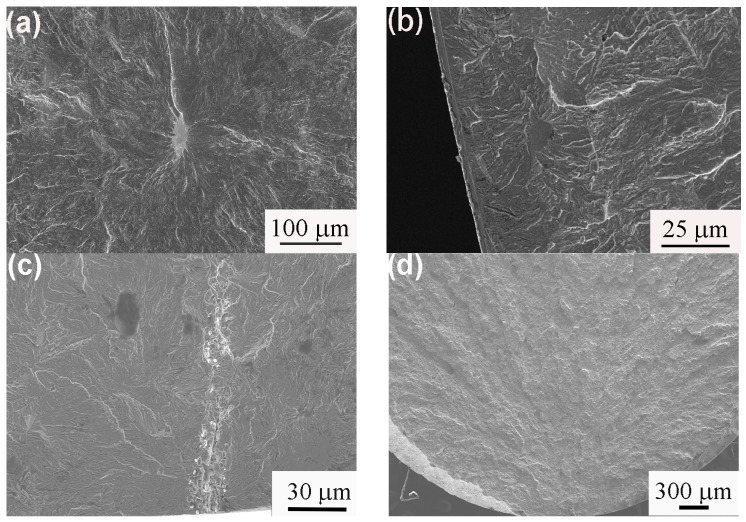
SEM fatigue fracture surface morphologies of isothermally-transformed samples. (**a**) A3 sample (1.4 GPa, 84,385 cycles), fracture initiate from MnS inclusion in sample interior; (**b**) A3 sample (1.6 GPa, 6206 cycles), fracture initiate from MnS inclusion in sample subsurface; (**c**) A1 sample (1.4 GPa, 61,429 cycles), fracture initiate from string of alumina inclusions on sample surface; (**d**) A1 sample (1.5 GPa, 9287 cycles), fracture initiated at or in close proximity to sample surface.

To investigate the fatigue fracture behavior and mechanism, the fracture surfaces were examined with SEM. The typical SEM images of the fatigue crack origins are presented in Figure 5. Two types of crack origins, such as inclusions (Figure 5a–c) and the surface or in close proximity to surface of the sample (Figure 5d), were observed. The inclusions were identified as MnS (Figure 5a,b) and alumina (Figure 5c) through EDS analysis.

**Figure 6:**
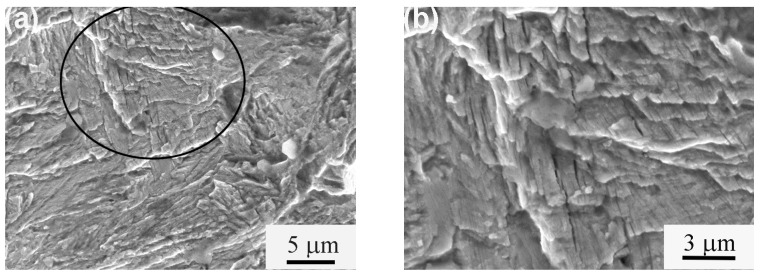
SEM image of fatigue fracture surface in crack propagation region of A3 sample following fatigue testing with maximum stress of 1.5 GPa and at 18,316 cycles: (**a**) Low magnification; (**b**) High magnification of the black circle.

The extensive SEM examination of the fatigue fracture surface in the crack propagation region demonstrated that secondary cracks existed in the fracture surface in an almost parallel arrangement. Figure 6 presents a typical SEM image of the fatigue fracture surface in the crack propagation region of the A3 sample subsequently to fatigue testing, with the maximum stress of 1.5 GPa and at 18,316 cycles. Figure 6b was the high magnification of the black circle in Figure 6a. The secondary cracks in the fracture in the almost parallel arrangement were also observed in the authors’ previous research [11]. The formation of the secondary cracks could relieve the triaxial stress conditions and stress concentration at the crack tip as well as blunt the crack tip, which could enhance the resistance to crack propagation. The formation of the secondary cracks during the fatigue crack propagation might be associated with the ferrite/austenite interfaces in the parallel arrangement. When the fatigue crack front propagated close to the interface, the phase transformation of austenite to martensite was induced by severe stress concentration at the crack tip, which could result in a high volume expansion and shear strain. Therefore, the interfaces were weakened and the secondary cracks were readily formed along the interfaces.

**Figure 7:**
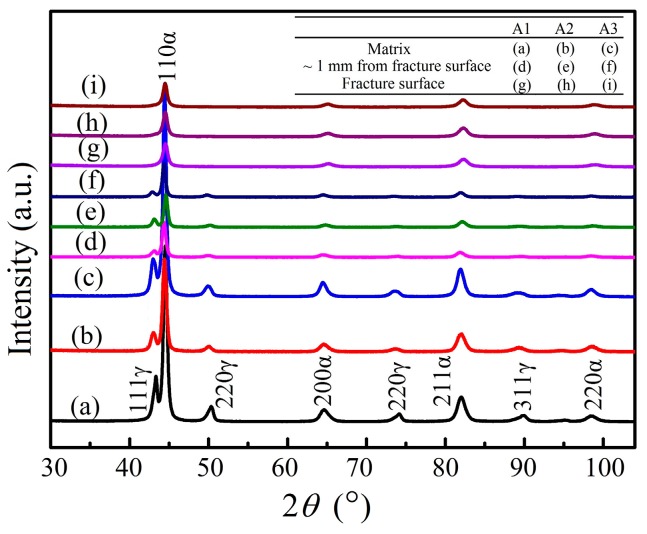
XRD patterns obtained in matrix, fatigue fracture surface and nearby fracture surface of (**A1**) (1.5 GPa, 8566 cycles); (**A2**) (1.5 GPa, 10,970 cycles) and (**A3**) (1.6 GPa, 12,254 cycles) samples.

In order to explore the retained austenite behavior during fatigue testing, XRD analysis was performed in the matrix, the fracture surface and the nearby fracture surface (~1 mm distance from surface) of the fatigue samples. The XRD patterns are presented in Figure 7. The XRD peaks of ferrite (α) and retained austenite (γ) were present in the XRD patterns of the matrix and the nearby fracture surface, while the retained austenite peaks were hardly observed in the XRD patterns of the fracture surface. The VRA was determined by the method proposed in reference [15] and the results are presented in Table 2. Compared to the matrix, the reduction of VRA in the nearby fracture surface was nearly negligible, suggesting that the retained austenite was stable under loading. In contrast, almost all retained austenite was transformed into martensite in the fracture surface. This meant that the transformation of retained austenite into martensite was induced by the stress concentration or plastic deformation at the front of the cracks, during the fatigue crack propagation or during fracture.

**Figure 8:**
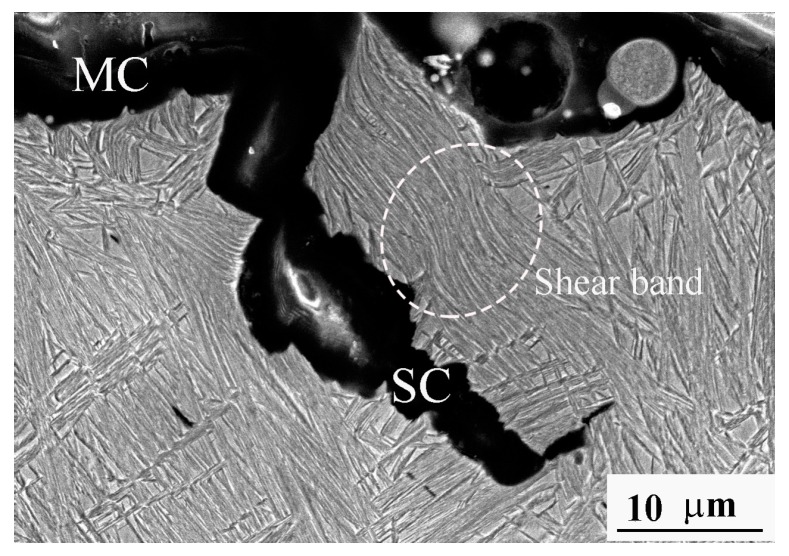
SEM fractograph of microstructure near main crack (**MC**) and secondary crack (**SC**) fracture surfaces of the A2 sample following fatigue break at maximum stress of 1.3 GPa and 49,168 cycles.

To understand the propagation behavior of the main and secondary cracks, the SEM examinations of the microstructure near the fatigue fracture surface were executed in the section along the crack propagation direction and normal to the fracture surface. Figure 8 presents the typical SEM fractograph of the A2 sample following fatigue testing at the maximum stress of 1.3 GPa and at 49,168 cycles. It could be observed that the lath microstructure, such as the nanostructured bainite, existed in the A2 sample. It was also observed that plastic deformation occurred in the near fracture surface layer of both the main crack (MC) and the secondary crack (SC); even the shear band was formed. The plastic deformation induced approximately all the retained austenite transformation into martensite, which was proved by the aforementioned XRD results. In Figure 8, one can see that a majority of the cracks were seen to develop perpendicularly to the lath structure, which was different from Figure 6. This may be attributed to the fact that the crack’s propagation may relate to the crystallographic connectivity between the groups of bainite/martensite laths [16]. The high plastic deformation in the crack propagation path caused the transformation of retained austenite to martensite at the crack tip during crack propagation, resulting in transformation-induced plasticity along with a strength increase at the crack front, which was positive to the fatigue limit increase.

## 4. Conclusions

Nanostructured bainite was prepared through high-C, Si–Al-rich steel by isothermal transformation at 220, 240 and 260 °C, which resulted in the ultrahigh ultimate tensile strength of 1925–2156 MPa. Through the data extrapolation, measured under the maximum cycle number of 10^5^, the push–pull fatigue limits at 10^7^ cycles were estimated as 889, 877 and 710 MPa for the samples isothermally transformed at 220, 240 and 260 °C, respectively. Both the interior inclusion and the sample surfaces could be the fatigue crack origins. During the fatigue crack propagation, a high amount of secondary cracks were generated in a parallel arrangement; also, apparent plastic deformation occurred in the near-fracture surface layer, which induced approximately all retained austenite to transform into martensite.

## Figures and Tables

**Table 1:** Mechanical properties of isothermally-transformed samples.

Sample	Transformed Temperatre (°C)	Hardness (HV)	UTS (MPa)	YS (MPa)	El (%)
A1	220	633	2156	1880	12.7
A2	240	617	2025	1495	15.0
A3	260	587	1925	1359	20.9

**Table 2:** VRA in matrix, fracture surface and nearby fracture surface of fatigue fracture samples through XRD (vol %).

Sample	Matrix	Nearby Fracture Surface	Fracture Surface
A1 (1.5 GPa, 8566 cycles)	~20.1	~18.4	Approximately 0
A2 (1.5 GPa, 10,970 cycles)	~22.4	~21.9	Approximately 0
A3 (1.6 GPa, 12,254 cycles)	~24.4	~23.6	Approximately 0
